# Posttraumatic Stress Disorder in the *DSM-5*: Controversy, Change, and Conceptual Considerations

**DOI:** 10.3390/bs7010007

**Published:** 2017-02-13

**Authors:** Anushka Pai, Alina M. Suris, Carol S. North

**Affiliations:** 1 VA North Texas Health Care System, 4500 S. Lancaster Road, 116A, Dallas, TX 75216, USA; alina.suris@va.gov; 2 Department of Psychiatry, The University of Texas Southwestern Medical Center, Dallas, TX 75390-8828, USA; Carol.North@utsouthwestern.edu; 3 Metrocare Services, Dallas, TX 75247-4914, USA

**Keywords:** posttraumatic stress disorder, psychiatric diagnosis, diagnostic criteria, nosology, trauma, *DSM-5*

## Abstract

The criteria for posttraumatic stress disorder PTSD have changed considerably with the newest edition of the American Psychiatric Association’s (APA) *Diagnostic and Statistical Manual of Mental Disorders* (*DSM-5*). Changes to the diagnostic criteria from the *DSM-IV* to *DSM-5* include: the relocation of PTSD from the anxiety disorders category to a new diagnostic category named “Trauma and Stressor-related Disorders”, the elimination of the subjective component to the definition of trauma, the explication and tightening of the definitions of trauma and exposure to it, the increase and rearrangement of the symptoms criteria, and changes in additional criteria and specifiers. This article will explore the nosology of the current diagnosis of PTSD by reviewing the changes made to the diagnostic criteria for PTSD in the *DSM-5* and discuss how these changes influence the conceptualization of PTSD.

## 1. Introduction

Posttraumatic stress disorder (PTSD) has attracted controversy since its introduction as a psychiatric disorder in the third edition of the American Psychiatric Association’s (APA) *Diagnostic and Statistical Manual of Mental Disorders* (*DSM-III*) in 1980 [1]. With each revision of the *DSM*, the criteria for PTSD have changed substantially [2]. Following publication of the fourth edition of the *DSM* (*DSM-IV*) in 1994 [3], PTSD experts criticized the criteria extensively, proposing myriad ways to address the problems they identified [4,5,6,7]. The literature accumulating during this time presented various polemical arguments concerning the definition of trauma and even questioning the need for it in the definition of PTSD [5,6,8], which and how many symptoms to include in the PTSD criteria and how they should be grouped [8], and even whether PTSD is a valid diagnosis at all [9]. Although the subsequent *DSM-IV* text revision edition of the manual (*DSM-IV-TR*) revised the text accompanying the criteria, the diagnostic criteria for PTSD did not change in this version. Therefore, this article will refer to these two versions together as *DSM-IV/-TR*. 

The fifth edition (*DSM-5*) of the criteria required seven years of planning, six years of actual work group activity, and a year to finalize the materials for publication and obtain the approval of the APA Assembly and Board of Trustees. The revision efforts included an extensive review of literature, secondary analyses, professional presentations and town halls, vigorous debates among trauma experts and nosologists, and rounds of public and professional reviews of the proposed criteria [10,11]. This was described as a “very conservative approach” [12] (p. 548), with the appreciation that because important clinical and scientific consequences could result from any modifications to the diagnostic criteria, the work group needed to have very strong evidence before making any changes. Regardless, the changes in the diagnostic criteria for PTSD from *DSM-IV/-TR* to *DSM-5* were substantial.

This article will explore the nosology of the current diagnosis of PTSD. Specifically, it will critically examine the *DSM-5* diagnostic criteria for PTSD, review changes in the criteria made in the *DSM-5*, and consider how the criteria shape current conceptualizations of PTSD. 

## 2. Diagnostic Classification of PTSD

Perhaps the most substantial conceptual change in the *DSM-5* for PTSD was the removal of the disorder from the anxiety disorders category. Considerable research has demonstrated that PTSD entails multiple emotions (e.g., guilt, shame, anger) outside of the fear/anxiety spectrum [13,14], thus providing evidence inconsistent with inclusion of PTSD with the anxiety disorders. In the *DSM-5*, PTSD was placed in a new diagnostic category named “Trauma and Stressor-related Disorders” indicating a common focus of the disorders in it as relating to adverse events. This diagnostic category is distinctive among psychiatric disorders in the requirement of exposure to a stressful event as a precondition. Other disorders included in this diagnostic category are adjustment disorder, reactive attachment disorder, disinhibited social engagement disorder, and acute stress disorder. This is the only diagnostic category in the DSM*-5* that is not grouped conceptually by the types of symptoms characteristic of the disorders in it. 

## 3. Criterion A: Exposure to Trauma

PTSD begins with criterion A, which requires exposure to a traumatic event. Criterion A is not only the most fundamental part of the nosology of PTSD, but also its most controversial aspect [12]. Some trauma experts criticized criterion A in the *DSM-IV* as too inclusive [5,6,15] and warned that this change had the potential to promote “conceptual bracket creep” [16] or “criterion creep” [17]. Some authors questioned the value of criterion A altogether [8,18,19], even suggesting that it should be abolished [8]. Criterion A was retained in the *DSM-5*, but it was modified to restrict its inclusiveness.

Not all stressful events involve trauma. The *DSM-5* definition of trauma requires “actual or threatened death, serious injury, or sexual violence” [10] (p. 271). Stressful events not involving an immediate threat to life or physical injury such as psychosocial stressors [4] (e.g., divorce or job loss) are not considered trauma in this definition.

The *DSM-5* has clarified and narrowed the types of events that qualify as “traumatic”. The ambiguous *DSM-IV/-TR* term “threat to physical integrity” [3] (p. 427) was removed from the definition of trauma in the *DSM-5*. Medically based trauma is now limited to sudden catastrophe such as waking during surgery or anaphylactic shock. Non-immediate, non-catastrophic life-threatening illness, such as terminal cancer, no longer qualifies as trauma, regardless of how stressful or severe it is. Medical incidents involving natural causes, such as a heart attack, no longer qualify (with the stated exception of life-threatening hemorrhage in one’s child, as described in the text accompanying the criteria). This seemingly minor revision of the definition of medically based trauma appears to have had a substantial influence on PTSD findings. A *DSM-IV/DSM-5* comparison study conducted by Kilpatrick and colleagues [20] using highly structured self-report inventories demonstrated that 60% of PTSD cases that met *DSM-IV* but not proposed *DSM-5* PTSD criteria were excluded from the *DSM-5* because the traumatic events involved only nonviolent deaths.

In addition to requiring the occurrence of a traumatic event, criterion A stipulates that the individual must have had a qualifying exposure to the trauma [2]. In other words, trauma is necessary, but it is not sufficient for consideration of PTSD without a qualifying exposure to that trauma. The *DSM-IV/-TR* provided three qualifying exposure types: direct personal exposure, witnessing of trauma to others, and indirect exposure through trauma experience of a family member or other close associate. Although some critics had argued for removal of the third (indirect) type of exposure [5,6], the *DSM-5* retained all three types of exposure from the *DSM-IV/-TR*, now listed in the *DSM-5* as A1–A3. A fourth exposure type (A4) has been added: repeated or extreme exposure to aversive details of a traumatic event, which applies to workers who encounter the consequences of traumatic events as part of their professional responsibilities (e.g., military mortuary workers, forensic child abuse investigators).

*DSM-IV/-TR* used the phrase “experienced, witnessed, or was confronted with” [3] (p. 467) to refer to the three types of exposures that are now listed and explicitly defined respectively as A1–A3 in the *DSM-5*. The ambiguous *DSM-IV/-TR* term “confronted with”, in apparent reference to indirect exposure through close associates, has been completely removed from the definition of exposure to trauma in the *DSM-5*. The *DSM-IV/-TR* did not specify whether witnessed exposures had to be in person, or whether media reports could constitute a witnessed exposure. The *DSM-5* has now clearly required the witnessing of trauma to others to be “in person” [10] (p. 271). Exposure through media is further narrowed in the *DSM-5* by specifying that “criterion A4 does not apply to exposure through electronic media, television, movies or pictures unless it is work-related” [10] (p. 271). These specific changes to the criteria defining trauma and qualifying exposures to it have important potential ramifications for the assessment and estimation of PTSD prevalence in real-life settings. For example, using the unspecified *DSM-IV/-TR* definition of witnessed trauma exposure, research studies counted media reports as trauma exposures, permitting nearly anyone living in the United States of America to be trauma-exposed in the 11 September 2001 terrorist attacks [5]. The nationwide incidence of “probable PTSD” related to the disaster was thus reported as 4% of the population [21], constituting an estimated total burden of 11 million cases [2]. The consequences of imprecise definitions of trauma and exposure to it are particularly extensive when large populations with non-qualifying trauma exposures are considered trauma-exposed for the purposes of measuring symptoms. Careful application of *DSM-5* criteria in the future can avert substantial inaccuracies in the estimation of PTSD prevalence.

*The DSM-5* removed the subjective personal response of “intense fear, horror, or helplessness” that had been added to criterion A in the *DSM-IV*. The requirement of a subjective response as part of the trauma criterion created a serious conceptual error by conflating the subjective experience of trauma with objective exposure to the traumatic event [4]. The personal response to trauma exposure, including posttraumatic symptoms, needs to be separated from the definition of trauma exposure for conceptual clarity [2]. In agreement with North and colleagues, McNally [5] (p. 598) recommended the elimination of criterion A2, arguing: “In the language of behaviorism, it confounds the response with the stimulus. In the language of medicine, it confounds the host with the pathogen”. The decision to remove criterion A2 from the *DSM-5*, however, was instead based on two specific research findings: (1) the requirement of a subjective response would exclude individuals who did not endorse fear, helplessness, or horror during the traumatic event, yet met the rest of the diagnostic criteria for PTSD [22,23], especially military personnel [2,24]; (2) the subjective response does not add predictive ability to the objective definition [22,25].

Exposure to trauma is the foundation for the rest of the criteria that comprise the diagnosis of PTSD [4,12,16,26]. Breslau et al. [27] emphasized that the link between PTSD symptoms and exposure to a traumatic event is what makes the diagnosis of PTSD a distinct disorder. They posed the question, “Without exposure to trauma, what is posttraumatic about the ensuing syndrome?” [27] (p. 927). North et al. [4] whimsically added that without exposure to trauma, a syndrome following a nontraumatic stressor might more appropriately be named “poststressor stress disorder” and one associated with no identified stressor called “nonstressor stress disorder”.

## 4. The Symptom Criteria

PTSD symptoms are conditionally linked to trauma exposure. Almost all other disorders in the *DSM* criteria are defined based on their characteristic symptoms, and thus the conditional nature of PTSD creates complexity not encountered in other disorders. According to the current diagnostic criteria, assessment of PTSD symptoms is appropriate only if criterion A is met, i.e., the individual has had a qualifying exposure to a requisite trauma. Without this trauma exposure, psychiatric symptoms reported by an individual would not qualify as PTSD symptoms. Each symptom must be anchored to the traumatic event through a temporal and/or contextual relationship [4]. The *DSM-5* stipulates that to qualify, the symptoms must begin (symptom criteria B and C) or worsen (symptom criteria D and E) after the traumatic event. Even though the symptoms must be linked to a traumatic event, this linking does not imply causality or etiology. Hence, the diagnostic criteria for PTSD are actually descriptive and agnostic toward etiology and therefore consistent with the generally descriptive and agnostic approach to defining psychiatric disorders in the American diagnostic system [4].

Revision of the PTSD symptom groups in the *DSM-5* relied on guidance from factor analytic research; however, findings from factor analytic studies examining the latent structure of PTSD symptoms to determine the most parsimonious symptom groupings have been inconsistent [28]. Additional factor analytic research has demonstrated substantial overlap of PTSD symptoms with symptoms of other disorders (especially depressive and anxiety disorders), inviting criticism of the validity of PTSD as a distinct disorder [15]. This factor analytic research has been limited, however, by use of self-report scales not anchoring symptoms to the traumatic event as defined by the diagnostic criteria for PTSD [4]. Factor analytic studies using data collected from structured diagnostic interviews that correctly link the symptoms contextually and temporally to the trauma exposure are needed to address these unresolved problems in the conceptualization of PTSD symptom criteria.

*The DSM-5* increased the number of symptom groups from three to four and the number of symptoms from 17 to 20. The *DSM-5* symptom groups are intrusion, avoidance, negative alterations in cognition and mood, and alterations in arousal and reactivity. To form the new group, *DSM-5* separated the avoidance and numbing symptoms into different groups. The two avoidance items from the *DSM-IV/-TR* avoidance/numbing group (criterion C) now comprise the *DSM-5* avoidance group (criterion C), and the numbing symptoms are now included with cognitive symptoms and mood symptoms in the negative cognition and mood group (criterion D). With this reorganization, at least one avoidance symptom is now required for an individual to meet diagnostic criteria in the *DSM-5*, in contrast to *DSM-IV/-TR* criteria which permitted a PTSD diagnosis even if no avoidance symptoms were endorsed.

Three new symptoms were added to the PTSD criteria in the *DSM-5*: persistent negative emotional state, persistent distorted cognitions about the cause or consequences of the trauma leading to blame of self or others, and reckless or self-destructive behavior. Reckless or self-destructive behavior was found to have low prevalence and poor factor loading in the *DSM-5* field trials, and this symptom was predominantly endorsed by the subgroup reporting the most severe symptoms [12,29]. The finding that only a limited subset of people endorsing severe symptoms acknowledged reckless or self-destructive behavior suggests that this symptom represents more a characteristic of a high symptom-endorsing subgroup and less a feature of the disorder itself. Reckless or self-destructive behavior was added as a symptom to the *DSM-5* criteria despite these research findings, because clinicians and researchers who observe it in the PTSD patient populations they work with believed it to represent a clinically important feature of the disorder [29]. Elsewhere it has been argued that inclusion of reckless/self-destructive behavior, persistent distorted cognitions, aggression toward others, and emphasis on dissociation have inserted cluster B personality features into PTSD, and that it may reflect selection biases based on observations of these features in specific subpopulations of PTSD, such as patients receiving psychiatric treatment [2]. Hoge and colleagues [30] criticized the added reckless/self-destructive behavior and negative emotional state symptoms as nonspecific to the psychopathology of PTSD and the persistent distorted cognitions symptom as over-pathologizing.

A number of *DSM-IV-TR* PTSD symptoms were revised in the *DSM-5* [2,30]. Some of the revisions involved minor wording changes (e.g., adding the word “involuntary” to “intrusive distressing recollections of the event”), and others were more foundational (e.g., “sense of a foreshortened future” reformulated as “persistent and exaggerated negative beliefs or expectations about oneself, others, or the world”; “restricted range of affect” changed to “persistent inability to experience positive emotions”). A study comparing *DSM-IV/-TR* and *DSM-5* symptom checklists for PTSD indicated that the changes to PTSD symptoms reflected in the checklists may have substantially altered the identification of PTSD cases [31].

## 5. Additional Criteria and Specifiers

A new set of PTSD criteria was added for children six years of age or younger to reflect their levels of development. The criteria for younger children do not have the “repeated or extreme exposure to aversive details of the traumatic event” exposure type, have only three symptom groups consisting of a total of 16 symptoms, have different symptoms grouped together compared to the adult symptom criteria, and indirect trauma exposure through a close associate is limited to a parent or care-giving figure. Additionally, intrusive memories in younger children do not have to appear distressing (as in play re-enactment) and nightmares do not have to be contextually based on the traumatic event. 

The acute and chronic PTSD specifiers were eliminated in the *DSM-5*, and the concept of delayed-onset PTSD was replaced with “delayed expression” defined as “the full diagnostic criteria are not met until at least 6 months after the event (although the onset and expression of some symptoms may be immediate)” [10] (p. 272). This is a diagnostic threshold definition of onset, using the point at which full diagnostic criteria are first met or last met as the point of onset or remission, respectively. Studies using repeated self-report symptom measures have used this method of determining onset and remission. Prior versions of *DSM* criteria have defined onset and remission of disorders as the point at which symptoms begin or end (i.e., a symptom-based definition of onset/remission), and structured diagnostic interviews have historically used this method of determining onset and remission [32]. The replacement of the previous PTSD onset criteria with the new delayed expression of onset definition in the *DSM-5* has effectively substituted a diagnostic threshold-based definition (which is found to yield a higher prevalence) for the historic symptom-based definition of onset [32]. This shift is destined to make it impossible to compare the onset of PTSD across studies using the new definition with historical estimates from previous research.

The *DSM-5* introduced a new dissociative features specifier to note the presence of associated persistent or recurrent depersonalization or derealization symptoms. This new feature of the disorder is a reflection of the focus of the *DSM-5* Trauma, PTSD, and Dissociative Disorders Sub-Work Group of the Anxiety Disorders Work Group committee that proposed the new PTSD criteria.

## 6. Conclusions

The diagnostic criteria for PTSD were substantially modified in the *DSM-5*, despite the revision process being described as “very conservative” by the work group [12]. The new changes in criterion A provide more conceptual clarity. Trauma exposure is objectively defined, and the subjective responses to trauma exposure (criterion A2) have been removed from criterion A, separating them from the trauma definition and confining them to the symptom criteria. This separation of the subjective response to trauma from the objective definition of trauma is an important advancement in the nosology of this conditionally-based disorder. The new criteria for trauma and exposure to it further limit the types of events that qualify as trauma for consideration of this disorder and more carefully define qualifying exposures to trauma.

Development of diagnostic criteria is an iterative process [4,33]. Additional research will be needed to validate this revision of the PTSD criteria, including study of descriptive characteristics, differential diagnosis, biological markers, and genetic factors [34]. Because the conditional definition of PTSD introduces complexity to its definition, it is paramount to study the criteria for PTSD with careful adherence to established criteria to permit testing of the criteria that are in use to inform future work.
